# The Impact of the COVID-19 Pandemic on the Spectrum of Performed Dental Procedures

**DOI:** 10.3390/ijerph18073421

**Published:** 2021-03-25

**Authors:** Kacper Nijakowski, Kornela Cieślik, Kacper Łaganowski, Dawid Gruszczyński, Anna Surdacka

**Affiliations:** 1 Department of Conservative Dentistry and Endodontics, Poznan University of Medical Sciences, 60-812 Poznan, Poland; annasurd@ump.edu.pl; 2 University Center of Dentistry and Specialized Medicine, 60-812 Poznan, Poland; k.cieslik@ucs.poznan.pl; 3 Student’s Scientific Group in Department of Conservative Dentistry and Endodontics, Poznan University of Medical Sciences, 60-812 Poznan, Poland; kacperloo111@gmail.com (K.Ł.); david.gruszczynsky@gmail.com (D.G.)

**Keywords:** COVID-19, SARS-CoV-2, pandemic, dentistry, dental procedures, conservative dentistry, dental surgery, tooth restoration, tooth extraction, dental services

## Abstract

The COVID-19 pandemic has significantly altered existing health care operations, including dentistry. The fear of SARS-CoV-2 infection and the need for increased protection measures have led to a reduction in the number of appointments and the range of performed procedures. Our study aimed to assess the impact of the COVID-19 pandemic (the pre-vaccine period) on the spectrum of performed dental services, with particular emphasis on the change in the proportion of conservative and surgical procedures. The patient base in the University Center of Dentistry and Specialized Medicine (Poznan, Poland) from two periods—pre-pandemic (1 February 2019–31 January 2020) and pandemic (1 February 2020–31 January 2021)—was analyzed. The number of dental services was standardized against the sum of all procedures in a given month. During the COVID-19 pandemic, the number of conservative procedures such as commercial restorations or filled canals has significantly decreased, while the number of surgical procedures has increased. The pandemic has undoubtedly affected the spectrum of dental procedures performed, especially in its acute phase. It is very important to return to performing conservative procedures and educating students in the former range while respecting all safety standards.

## 1. Introduction

*Coronaviridae* are positive-sense single-stranded RNA viruses. Betacoronavirus SARS-CoV-2 causes severe acute respiratory syndrome, named by the World Health Organization (WHO), COVID-19 [1]. This disease acronym has derived from the terms CO-rona VI-rus Disease and the year of identification-19 [2]. The outbreak began in late 2019 in Wuhan, China, and quickly spread beyond Asia to other continents. WHO first declared a Public Health Emergency of International Concern at the end of January 2020 and later announced the COVID-19 pandemic in March 2020 [1].

Last March, the New York Times published a schematic figure showing that dentists are the professional group that faces the highest risk of coronavirus exposition, ahead of general physicians and nurses [3]. For dentists, the primary goal should be the welfare of the patient, avoiding cross-infections. Therefore, deferring and suspending scheduled appointments is to protect patients from SARS-CoV-2 infection [4]. Despite their own anxieties and the expected financial consequences, dental professionals have reduced routine visits feeling morally obliged to prevent the development of the pandemic [5].

During the COVID-19 pandemic, it is particularly important for dentists to make appropriate clinical decisions as well as to educate patients to prevent potential complications properly. The key question should not be “to treat or not to treat?” but rather “how to treat?”. General recommendations provided for medical professionals need to be modified for the specific working conditions of dental practitioners [6].

In analyzing the impact of the COVID-19 pandemic on all aspects of life, including dental services, it is recommended to distinguish the pre-vaccine and post-vaccine periods. The introduction of the vaccine could become a key milestone that could prevent the next lockdowns. In the first phase of the pandemic, just after its outbreak, there was a significant reduction in dental visits in order to counteract the spread of the coronavirus [7]. It is worth noting that coronavirus infection may also be associated with oral symptoms, such as dysgeusia (taste disorders), xerostomia, or burning syndrome [8].

Dental professionals should follow the sanitary and epidemiological guidelines established by their country, in line with the three-step protection recommended by the WHO [9]. The most important steps in protecting and reducing contamination are considered to be patient triage, prescription of mouth rinses prior to dental treatment, hand hygiene for dentists and patients, personal protective equipment for dental practitioners, limitation of aerosol-producing procedures, and cleaning of potentially contaminated surfaces [10]. With regard to the use of antiseptic mouth-rinses, various recommendations are mentioned in the literature. It is suggested to gently gargle for 30 s in the oral cavity and 30 s in the back of the throat with: 1.5% or 3% H_2_O_2_ (15 mL), 0.2%, 0.4% or 0.5% povidone-iodine (9 mL), 0.12% chlorhexidine (15 mL), or 0.05% cetylpyridinium chloride (15 mL) [11]. Also, the use of anti-retraction dental handpieces with specially designed valves should be recommended as an extra preventive measure for cross-infection [12]. Chairside screening saliva tests for SARS-CoV-2 might be a good option to minimize the spread of the coronavirus in the office [13]. Despite the need for effective ventilation, the number of staff members in the surgery should be limited to a minimum [14]. Dentists treating children should observe the highest protection standards against infection, as children often have much milder symptoms than adults [15].

Various authors have attempted to classify dental procedures according to the risk of infection for the staff, as well as the patients. All agreed that the key factors influencing the possibility of SARS-CoV-2 infection during a dental visit are aerosol-generating procedures (especially with the use of the high-speed handpiece or the ultrasonic instruments) [16,17]. Bizzoca et al. [18] proposed the following classification according to the potential risk of infection. Radiological examination, topical treatment of dental hypersensitivity and caries prophylaxis, endodontic treatment of a single-rooted tooth with the rubber dam (in subsequent appointment after access cavity), extraction without rotary tools, and minor oral surgery (e.g., abscess incision) were assessed as low-risk procedures. In turn, among the high-risk procedures, tooth restoration using rotary tools, endodontic access cavity with rotary instruments, ultrasonic scaling, and surgical extraction with rotary tools were listed.

At the beginning of the COVID-19 pandemic in Poland, most dental practices voluntarily suspended their activities due to the lack of preparedness of the public and private sector in terms of handling sanitary procedures and availability of appropriate advanced personal protective equipment. This has led to an increase in anxiety among dentists and patients, thus decreasing the provision of dental services [19]. Most dentists reported closing their practices for 1–2 months. Typically, the decline in patient enrollment has been accompanied by an increase in the price of commercial dental services [20]. Interestingly, in the first weeks of the pandemic, Google searches for the phrase “toothache” (in Polish “ból zęba”) increased, while searches for the phrase “dentist” (in Polish “dentysta”) decreased significantly, in line with trends in other European countries such as Italy or the United Kingdom. This negative strong correlation started to reverse only at the turn of May and June 2020 [21].

Our study aimed to assess the impact of the COVID-19 pandemic (the pre-vaccine period) on the spectrum of performed dental services, with particular emphasis on the change in the proportion of conservative and surgical procedures.

## 2. Materials and Methods

The patient base in the University Center of Dentistry and Specialized Medicine (Poznan, Poland) from two periods—pre-pandemic (1 February 2019–31 January 2020) and pandemic (1 February 2020–31 January 2021)—was analyzed. Our University is one of the leading academic centers located in Greater Poland, the central region of the country. In Poland, the first wave of the COVID-19 pandemic started in March 2020, and the second wave in November 2020—each of them lasted about 2–3 months.

**Figure 1:**
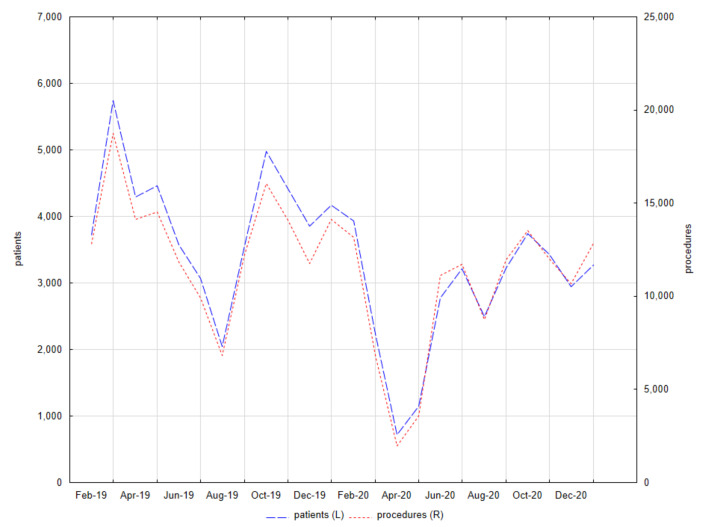
Patients and procedures in the University Center of Dentistry and Specialized Medicine (Poznan, Poland) from 1 February 2019 to 31 January 2021.

Patients admitted only in outpatient clinics that did not suspend their activities during the pandemic were selected, i.e., Clinic for Conservative Dentistry and Periodontology, Clinic for Oral Surgery, and Central Dental Clinic. The number of patients and provided services was determined by month—Figure 1.

Selected procedures in conservative dentistry with endodontics and dental surgery, both in children and adults, were analyzed in detail. The number of these individual services was standardized against the sum of all procedures in a given month. Comparisons were made between performed services in the pre-pandemic period and the pandemic period using the Mann–Whitney test, as well as the proportion of conservative and surgical procedures in the respective periods using the Wilcoxon test. The significance level was set at α = 0.05. All analyses were performed using Statistica 13.3 (Statsoft, Cracow, Poland). Graphs of the time dependence were made using the “time series” module.

## 3. Results

**Figure 2:**
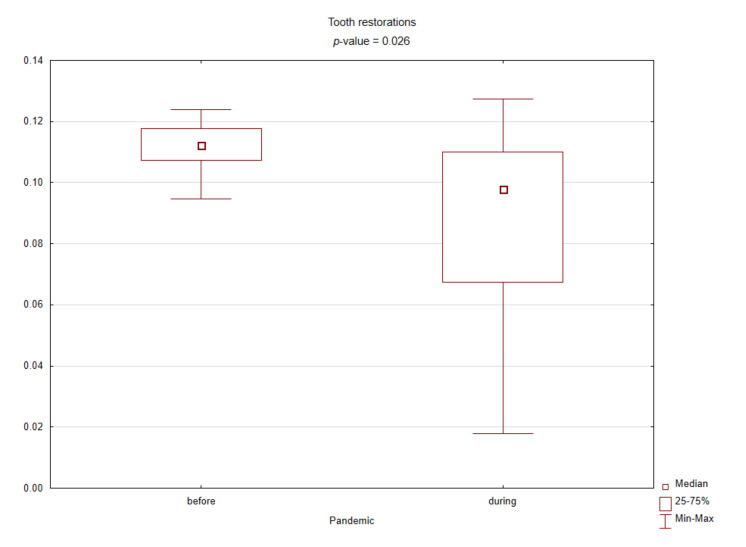
Box plot for the standardized number of tooth restorations before and during the COVID-19 pandemic (*p*-value < 0.05 according to the Mann–Whitney test).

**Figure 3:**
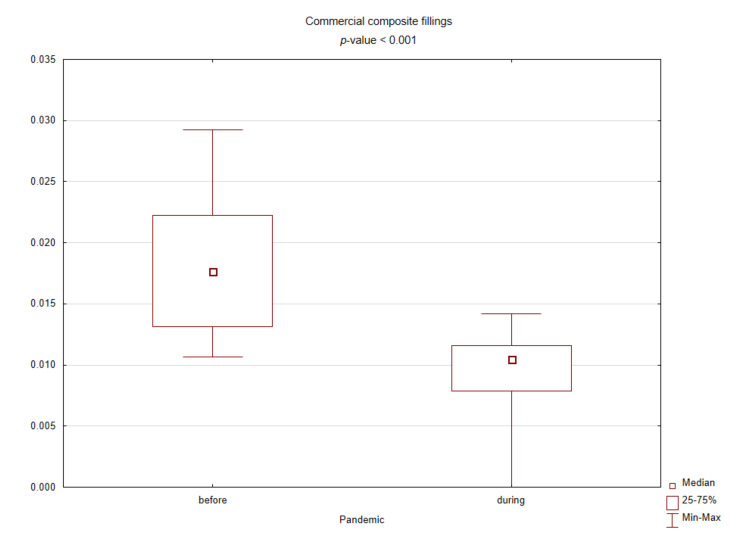
Box plot for the standardized number of commercial composite fillings before and during the COVID-19 pandemic (*p*-value < 0.05 according to the Mann–Whitney test).

**Figure 4:**
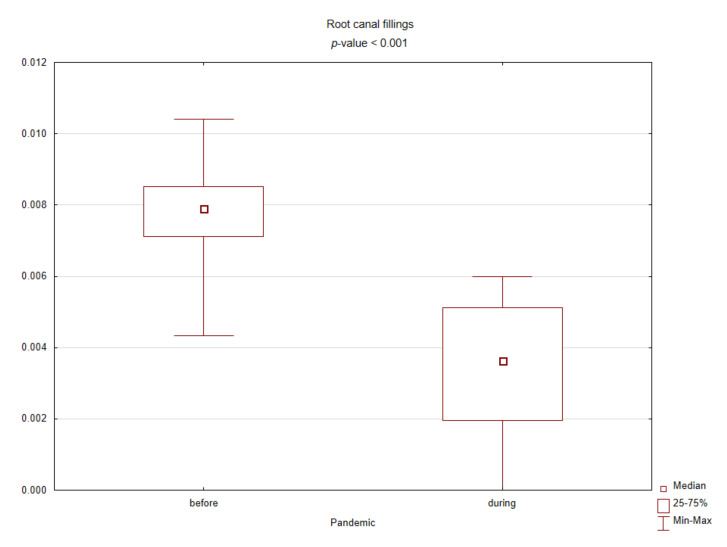
Box plot for the standardized number of root canal fillings before and during the COVID-19 pandemic (*p*-value < 0.05 according to the Mann–Whitney test).

**Figure 5:**
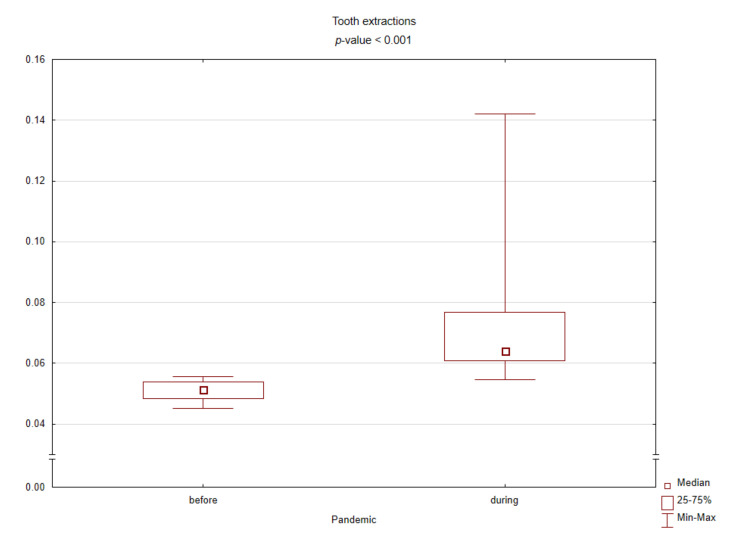
Box plot for the standardized number of tooth extractions before and during the COVID-19 pandemic (*p*-value < 0.05 according to the Mann–Whitney test).

Table 1 presents the results of comparing the spectrum of procedures performed before and during the pandemic based on standardized values against their sum in the given months. During the COVID-19 pandemic, the number of conservative procedures such as commercial restorations or filled canals has significantly decreased, while the number of surgical procedures has increased. Figure 2, Figure 3, Figure 4 and Figure 5 graphically show the differences between the study periods in terms of number of tooth restorations, number of commercial composite fillings, number of root canal fillings, and number of tooth extractions. The graphs clearly confirm the increase in the need for surgical services and decreased interest in conservative services during the pandemic period.

**Figure 6:**
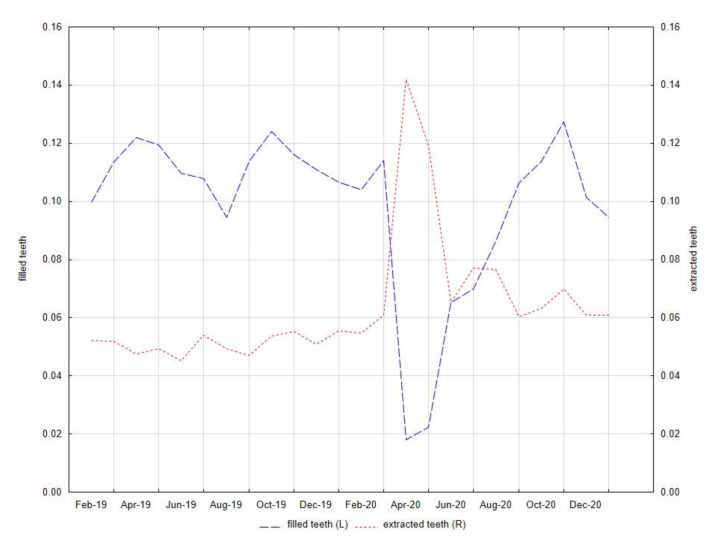
The standardized numbers of filled teeth and extracted teeth in the University Center of Dentistry and Specialized Medicine (Poznan, Poland) from 1 February 2019 to 31 January 2021.

Figure 6 shows how the number of services provided in the field of conservative dentistry (number of filled teeth) and dental surgery (number of extracted teeth) has changed over the study time. There is a clear advantage of surgical over conservative procedures in the spring of 2020 when the first wave of the COVID-19 pandemic took place in Poland. Before the pandemic, patients were far more likely to opt for conservative treatment, whereas in the pandemic more common surgical procedures eliminated this superiority—according to the Wilcoxon test, for comparison of filled and extracted teeth, *p*-values were 0.002 and 0.308, respectively, before and during the pandemic.

## 4. Discussion

The COVID-19 pandemic has taken a considerable toll on the health and well-being of doctors and other health professionals, often testing their professional competence to the limits [22]. However, maintaining the quality of dental services at a certain level influences patient satisfaction [23]. When analyzing dental patient satisfaction, the most important factors are competence and courtesy during the dental office visit, which are based on the interpersonal contact between the medical team and the patient. Patients, when assessing their satisfaction with the provided dental services, pay particular attention to the quality of the procedure. In the opinion of the respondents, the diagnosis of dental care in Greater Poland is good [24]. Moreover, attention should be paid to the occurrence of relations between patients’ expectations towards dental care and sociodemographic data [25].

The triage station has been operating in our Center since the beginning of the COVID-19 pandemic. At the acute phase of the pandemic, only emergency patients with a negative epidemic history were admitted. The recommended basic epidemiological history should include answers to three questions about direct contact with the SARS-CoV-2 suspected or positive person, recent travel to countries with a high prevalence of SARS-CoV-2 cases, and presence of respiratory symptoms such as cough or fever for at least two weeks [26]. According to de Almeida Barros Mourão et al. [27], up to three negative results are necessary for safe interpersonal contact between doctor and patient: the remote epidemiological interview, the temperature measurement before entering the office, and the quick test for SARS-CoV-2 infection. Spicciarelli et al. [28] suggested a multilevel evaluation (including oral, systemic, and psychological criteria) to manage dental emergencies following the COVID-19 pandemic. In addition to the epidemiological history, the following medical factors determining the need for urgent dental intervention should be considered during telephone triage: presence/absence of pain (and type of pain, if present), presence/absence of swelling (and localization and characteristics of swelling, if present), presence/absence of bleeding, as well as systemic diseases or medications and psychiatric or neurological disorders. Ayub and Alani [29], citing the NHS England guidelines, listed life-threatening emergencies (e.g., orofacial swellings causing airway restriction or breathing/swallowing difficulties), dento-alveolar trauma (including facial/oral laceration and/or dento-alveolar injuries such as fractures and luxations), severe dental or facial pain which cannot be controlled by the patient following self-help advice, as well as post-extraction bleeding which cannot be controlled by the patient with local measures as being among the conditions requiring urgent dental treatment. Suspected or positive SARS-CoV-2 patients should be admitted in special dental emergency centers.

Moreover, Peditto et al. [30] have divided urgent dental procedures into three categories. The first category of potentially life-threatening conditions requiring immediate intervention included uncontrolled bleeding and diffused soft tissue infection with intra-oral or extra-oral swelling. Among the second category conditions requiring 24-h intervention for pain relief were severe dental pain from pulpal inflammation, pericoronitis or third-molar pain, surgical post-operative osteitis, abscess or localized bacterial infection, tooth fracture resulting in pain or causing soft tissue trauma, dental trauma with avulsion/luxation, dental treatment required prior to critical medical procedures, final crown/bridge cementation if the temporary restoration is lost, broken or causing gum irritation, as well as biopsy of abnormal tissue. Extensive dental caries or defective restorations causing pain, suture removal, denture adjustment on radiation/oncology patients, denture adjustments or repairs when function impeded, replacing temporary filling on endo access openings in patients experiencing pain, and snipping or adjustment of an orthodontic wire or appliances piercing or ulcerating the oral mucosa were classified as the third category named “undeferrable treatments” (within a period longer than 24 h). In turn, routine dental treatment should be suspended until the acute phase of the pandemic is over. The authors recommended performing conservative and surgical procedures per quadrant. The number of admitted patients should be limited, and the maximum possible number of procedures should be performed per visit.

During the first months of the pandemic, the spectrum of dental procedures has significantly narrowed. In this period, our Center has seen a clear reversal of the previous proportions between conservative and surgical procedures, in favor of the latter. In emergency cases, pharmacological therapy should have been considered first, and if this was not possible, dental treatment should have been implemented, but in a manner limiting aerosol formation and thus the risk of contagion. Moreover, adequate time should be allowed between visits to ensure effective decontamination of the surgery [31]. At that time, dental societies recommended the chemomechanical cleaning of carious lesions with hand instruments instead of rotary ones. Similarly, for periodontal treatment, manual scaling should have been used instead of ultrasonic. In cases of symptomatic irreversible pulpitis, biological methods such as pulpotomy or pulpectomy should have been used by choice if possible. On the other hand, in patients with extensive destruction of tooth tissue and suffering a severe toothache, it was necessary to opt for extraction of the causative tooth in order to reduce the risk of infection, shorten the treatment time, and minimize repeat visits [32]. In case of excessive bleeding, multiple extractions, or other indications for wound supply, absorbable sutures should have been preferred. In radiological diagnostics, extraoral radiographs (such as panoramic X-ray or CBCT) should have been treated as an alternative to intraoral ones. For routine dental practice, N-95 or FFP2-standard masks should be applied. Moreover, the four-handed technique, high-volume saliva ejectors, and rubber dams were considered useful in minimizing the spread of generated aerosol [33].

For pediatric patients, specific guidelines were made to reduce aerosol-generating procedures and to use non-invasive and mini-invasive methods. Recommended biological treatment alternatives for asymptomatic teeth or with signs of reversible pulpitis included sealing, fluoride varnishing, and resin infiltration to arrest non-cavitated caries, indirect pulp capping, atraumatic restorative technique, interim therapeutic restorations, the Hall technique, and the application of Silver Diamine Fluoride. It has been advised to cancel all elective procedures, including those under general anesthesia and for medically compromised children [34]. In addition, patients with systemic diseases should require special dental care, even during pandemic periods. For example, patients with congenital bleeding disorders are at risk for uncontrolled bleeding after oral procedures [35].

In addition to a significant reduction in the range of dental services provided by our Center, there was a significant drop in the number of patients admitted at the start of the COVID-19 pandemic. During the first wave of the pandemic, limiting admissions to urgent cases only had financial repercussions for dental practices. Despite a decrease in total patient quantity, Chamorro-Petronacci et al. [36] observed significantly more patients with tooth pain in public sector units than in private ones. Schwendicke et al. [37] assessed the profound economic impact of the COVID-19 pandemic on dental offices. Practice owners might consider reorganization to reduce costs and maintain even minimal profitability. The constructed model showed that the longer the pandemic lasted, the greater the financial crisis facing dental clinics. The authors suggested that legislators should include financial assistance to dental practices to provide adequate protective measures, significantly increasing operational costs.

The pandemic quarantine undoubtedly influenced patients’ anxiety levels and their attitudes towards dental visits. Peloso et al. [38] observed that patients undergoing dental treatment were more willing to attend appointments. Moreover, orthodontic patients were the most concerned about prolonging treatment due to postponed visits. In contrast, patients not undergoing treatment reported seeing a dentist only for immediate tooth pain relief. The pandemic and its associated restrictions were also a source of concern for dental practitioners. Consolo et al. [39] conducted a psychological study in the region of northern Italy, one of the most involved during the most critical period of the COVID-19 pandemic. All surveyed dentists reported practice closure or a significant decrease in visits because many patients canceled appointments after the pandemic outbreak. Reduced work activity was accompanied by negative feelings such as concern (70.2%), anxiety (46.4%), and fear (42.4%). According to the GAD-7 (General Anxiety Disorder-7) evaluation, 9% of respondents demonstrated severe anxiety. The majority of worries were related to the current financial situation and the future of a professional career.

The COVID-19 pandemic also affected the number of admissions to maxillofacial surgery departments. As reported by Maffia et al. [40], the majority of surveyed institutions reduced the limits of procedures, particularly elective procedures. Only six surgical centers from Japan, Pakistan, Israel, Singapore, Poland, and Taiwan declared normal productivity, although the first two countries had high alert levels on the DORSCON Scale (“Disease Outbreak Response System Condition” Scale) The lack of precise and unified guidelines can result in unacceptably hazardous working conditions. However, Barca et al. [41] have tried to develop a facial pathology triage protocol to minimize the risk of SARS-CoV-2 infection to surgeons. In these authors’ department, patients were admitted mainly for trauma or non-differentiable oncological disease—respectively, 20 with fractures and 13 with cancerous tumors during the COVID-19 emergency period. Injuries mostly involved the mandible (7 patients), followed by orbital-maxillo-zygomatic complex [COMZ] (5 patients), orbital walls (4 patients), nasal bones (3 patients), and naso-orbital-ethmoid complex [NOE] (1 patient). Only 15 of the injured patients have been treated under general anesthesia with internal rigid fixation through plates and screws (all patients with mandibular, orbital and NOE fractures, 3 with COMZ fractures). The remaining 5 patients were referred to outpatient clinics due to compound injuries not requiring surgical intervention. Among oncological patients, 7 of them have been operated under general anesthesia (1 with oral squamous cell carcinoma, 5 with squamous skin cancer and 1 with mucoepidermoid carcinoma in submandibular gland). The surgical techniques were chosen on the basis of careful clinical assessment to simplify the procedure and reduce the operating duration.

Many countries have introduced telephone and internet consultations for patients with various symptoms [42]. Unfortunately, most dental disorders cannot be fully diagnosed in this way, as well as properly and effectively managed. Therefore, the quick return to relatively safe dental care, as well as dental education, is so important. Nevertheless, three modes of remote oral diagnosis are described in dentistry [43]. Teleorientation enables to perform screening, instruct and refer patients suspected or positive for SARS-CoV-2 to the face-to-face appointment if needed. Telemonitoring is visual monitoring of oral lesions through photographs sent by patients. Unfortunately, they can sometimes have insufficient resolution. Lastly, teleconsultation involves information exchange between professionals in terms of diagnosis and therapy. Machado et al. [44] reported the example of oral telediagnostics. The 49 year-old female patient with controlled diabetes presented pinky-purple symptomatic nodule lesions affecting the oral mucosa, associated with purple spots on the skin. The dentist made her images with a brief description available by WhatsApp, and she was advised to order a blood examination due to suspected Idiopathic Purpura. The severe thrombocytopenia was confirmed, and the patient was referred to the hospital in order to the systemic steroid therapy.

## 5. Conclusions

The COVID-19 Pandemic has undoubtedly affected the spectrum of dental procedures performed, especially in its acute phase. The quantity of emergency and more invasive surgical procedures has significantly increased, while planned conservative procedures have decreased. In subsequent months, the reduced number of patients was compensated by a higher number of procedures performed per visit. Thanks to the development of safe working principles and the introduction of vaccination, it becomes possible to slowly return to carrying out the former number of conservative procedures, including endodontic ones, and to continue the dental education in a hybrid model instead of a remote one.

## Figures and Tables

**Table 1:** Medians and quartiles for standardized numbers of selected procedures performed before and during the COVID-19 pandemic in the University Center of Dentistry and Specialized Medicine (Poznan, Poland) from 1 February 2019 to 31 January 2021.

	Before Pandemic	During Pandemic	
Procedure	M (Q1–Q3)	M (Q1–Q3)	*p*-Value
dental examination	0.171 (0.167–0.177)	0.172 (0.160–0.188)	0.840
periapical X-ray	0.043 (0.040–0.050)	0.044 (0.039–0.050)	0.977
panoramic X-ray	0.029 (0.025–0.030)	0.027 (0.024–0.031)	0.795
*Conservative dentistry*			
temporary filling	0.019 (0.019–0.022)	0.018 (0.016–0.022)	0.260
single-surface filling	0.042 (0.038–0.047)	0.038 (0.019–0.043)	0.141
two-surface filling	0.040 (0.033–0.043)	0.032 (0.021–0.037)	0.019 *
multi-surface filling	0.009 (0.007–0.011)	0.007 (0.006–0.008)	0.061
refunded restoration	0.075 (0.066–0.078)	0.068 (0.038–0.076)	0.285
commercial restoration	0.018 (0.013–0.022)	0.010 (0.008–0.012)	<0.001 *
filled deciduous teeth	0.022 (0.019–0.023)	0.020 (0.017–0.022)	0.312
filled permanent teeth	0.091 (0.084–0.095)	0.078 (0.046–0.088)	0.046 *
total filled teeth	0.112 (0.107–0.118)	0.098 (0.067–0.110)	0.026 *
*Endodontics*			
intervention procedure	0.008 (0.007–0.010)	0.010 (0.009–0.011)	0.078
intracanal dressing	0.003 (0.003–0.004)	0.003 (0.003–0.004)	0.564
root canal filling	0.008 (0.007–0.009)	0.004 (0.002–0.005)	<0.001 *
*Periodontology*			
scaling (quadrant)	0.111 (0.104–0.118)	0.105 (0.070–0.123)	0.507
curettage (quadrant)	0.006 (0.006–0.007)	0.005 (0.003–0.007)	0.194
gingival pocket rinsing	0.012 (0.010–0.013)	0.011 (0.008–0.015)	0.885
oral lesions application	0.004 (0.003–0.005)	0.004 (0.002–0.004)	0.341
*Dental surgery*			
abscess drainage	0.001 (0.001–0.002)	0.002 (0.002–0.003)	0.002 *
intra-alveolar dressing	0.023 (0.019–0.027)	0.031 (0.025–0.034)	0.005 *
single-rooted tooth extraction	0.010 (0.009–0.010)	0.011 (0.010–0.011)	0.089
multi-rooted tooth extraction	0.012 (0.011–0.013)	0.014 (0.013–0.018)	0.006 *
intra-alveolar extraction	0.023 (0.020–0.027)	0.029 (0.027–0.034)	0.002 *
extra-alveolar extraction	0.003 (0.002–0.003)	0.005 (0.004–0.009)	0.002 *
impacted tooth extraction	0.003 (0.003–0.004)	0.007 (0.005–0.008)	0.003 *
total extracted teeth	0.051 (0.048–0.054)	0.064 (0.061–0.077)	<0.001 *

* significant difference for *p*-value < 0.05 according to the Mann–Whitney test.

## Data Availability

Not applicable.
